# National review of maternity services 2008: women influencing change

**DOI:** 10.1186/1471-2393-11-53

**Published:** 2011-07-16

**Authors:** Meredith J McIntyre, Karen Francis, Ysanne Chapman

**Affiliations:** 1 School of Nursing & Midwifery, Monash University, Peninsula Campus, Frankston, Australia; 2 School of Nursing & Midwifery, Monash University, Gippsland Campus, Churchill, Australia; 3 School of Nursing & Midwifery, Central Queensland University, Mackay Campus, Australia

## Abstract

**Background:**

In 2009 the Australian government announced a major program of reform with the move to primary maternity care. The reform agenda represents a dramatic change to maternity care provision in a society that has embraced technology across all aspects of life including childbirth.

**Methods:**

A critical discourse analysis of selected submissions in the consultation process to the national review of maternity services 2008 was undertaken to identify the contributions of individual women, consumer groups and organisations representing the interests of women.

**Results:**

Findings from this critical discourse analysis revealed extensive similarities between the discourses identified in the submissions with the direction of the 2009 proposed primary maternity care reform agenda. The rise of consumer influence in maternity care policy reflects a changing of the guard as doctors' traditional authority is questioned by strong consumer organisations and informed consumers.

**Conclusions:**

Unified consumer influence advocating a move away from obstetric -led maternity care for all pregnant women appears to be synergistic with the ethos of corporate governance and a neoliberal approach to maternity service policy. The silent voice of one consumer group (women happy with their obstetric-led care) in the consultation process has inadvertently contributed to a consensus of opinion in support of the reforms in the absence of the counter viewpoint.

## Background

In 2009 the Australian government has announced a major program of reform with the move to primary maternity care. Primary maternity services are based on the understanding that 85% of pregnant women are capable of giving birth safely with minimal intervention [1-4]. It is argued the removal of uncomplicated childbirth from routine obstetric influence will reduce the numbers of women receiving expensive obstetric care and interventions in the absence of clinical need [5]. The reforms are expected to provide the right balance between primary level care and access to appropriate levels of medical expertise as clinically required [6]. In announcing the reforms the government has responded to a decade of pressure from numerous reports, commissions and inquiries recommending wide scale change in how maternity care is to be delivered in Australia [7-16].

Implementation of the primary maternity care reforms will not be without challenges. Australia is a society that has embraced the introduction of high technology across all aspects of life including childbirth. We live in a 'no risk' society [17] in which technology-intensive childbirth is equated with high standards of care employed in the best interest of women and babies [18]. Families have come to fear birth justifiably in light of the messages they receive from some health professionals, media and social networks reinforcing the belief that 'childbirth is inherently risky' [19-21], a 'difficult' process from which mothers and babies need to be rescued [22]. Parents know that they are not the experts in pregnancy and birth and that the consequences of any problems that may arise can be catastrophic [18].

In contrast the primary maternity care reform agenda represents a dramatic move away from expensive high tech specialist services [3]. It is clear from the findings of the national review of maternity services undertaken in 2008, that the views of women and families have been integral to the consultation process. In the interests of successful implementation of the reforms it is important to determine the extent of general public support for the changes proposed. The majority of consumers of maternity services are a transitory population remaining involved as a recipient for a short time before moving on to other interests as their children grow and they return to the workforce [18]. These women are represented in the 96.9% of births recorded in Australia in 2008 that took place in hospital birth suite settings with a further 2.2% who gave birth in the few remaining birth centres in operation across the country [23]. A small number of consumers [24] passionate to their cause join consumer movements such as the maternity coalition and home birth Australia, organisations dedicated to promoting natural childbirth. It is clear that not all women nor their friends and family share the ideal of an intervention-free birth [17,19]. It is argued that women's expectations of birth are situated at opposite ends of the spectrum. They either perceive the birthing experience to be a normal, natural process that women can or should achieve themselves or to be a pathologically hazardous event, fraught with risk and danger to be feared and surrendered to medical control [25]. It is important to determine whose views were heard in the consultation process and whose views were not?

## Methods

### Research design

A critical analysis of women's submissions to the national review of maternity services 2008 was undertaken for the purpose of identifying the views of women whose influence contributed to the direction of the Australian maternity care reform agenda. The analysis was informed by Foucault's work related to medical practice, the body and issues of power within these discourses [26]. Discourses according to Foucault (1972) are bodies or fields of knowledge that contain all the possible statements about what is known or said about a thing [27]. Pertinent to this study are the questions; what is known and what can be said on the topic of what women want in their maternity care and the spokespersons created within the discourse. A discourse can be viewed as a total system of knowledge that makes a multitude of true statements possible whilst articulating a particular truth and then maintaining the effects of that truth [28]. A discourse always produces power as well as knowledge, the effects of which act to maintain institutions such as medicine, obstetrics or midwifery [28].

Critical analysis of discourse as a methodology was chosen for this study because language takes on greater significance in the arena of providing and consuming services [29]. Maternity care provider groups incorporating obstetrics and midwifery have a long history involved in professional power struggles whilst consumer groups also contain polarised elements making an analysis of language used to influence public attitudes an appropriate choice. Critical analysis of discourse provides insight into how bodies of knowledge are used or controlled by groups with vested interests such as those in maternity care delivery [30]. The ability to generate interpretive claims with regard to the desired consequences of controlling knowledge [31], combined with the ability to critically examine power relationships constituted by discourse [32] are relevant to this study.

### Theoretical Framework

Critical social theory has greatly influenced the development of CDA bringing together a wide variety of critical social theories including the way power is conceptualized [33]. Through his work Foucault has influenced the understanding that discourse is 'an instrument in the social construction of reality'[29]. CDA views discourse as central to the functioning of power in social processes and the reproduction of power in a given situation, and to understand the processes of power and how these processes use discourses to achieve power. It is crucial that language is not analyzed out of context, but is situated within the specific context of social practices of which it is a part [34]. This necessitates an approach that takes in the broad context of the text in addition to the specific words used.

### Ethical Considerations

All research data consisted of written submissions to the national review of maternity services in 2008 published on the Australian government website. This data is freely available and permission is not required to access this data source. Personal information was not retrieved from any source and there was no participant involvement.

### Data Collection

Selected submissions to the national review of maternity services in 2008 from individual women or groups representing women were examined. The consultation process of the national review included 832 submissions from key stake holders, the personal stories of individual women made up 407 of the submissions received [5]. A significant proportion of these submissions (53 per cent) were from women who had personally experienced homebirth, a population that represents 0.9% of total births in Australia in 2008 [23]. Selected submissions were chosen to ensure adequate representation from the 96.9% of women who gave birth in hospitals and the 2.2% who gave birth in birth centres in addition to the 0.9% who gave birth at home [23]. A total of thirty-six submissions were examined and analysed. Criteria for submission selection are outlined in Table 1. Submissions selected included: individual women (n = 16), and groups representing women (n = 18) and consumer lobby groups (n = 2).

**Table 1:** Selected Submissions to the National Review of Maternity Services.

Women & Consumer Key Stakeholders	Rationale for inclusion	Submission Numbers:
Maternity Coalition (MC).	*National consumer organisation (natural birth)*	354
Multicultural Centre for Women's Health.	*Organisation representing women from multicultural backgrounds*	190
National Foundation for Australian Women.	*National organisation representing women*	220
National Rural Women's Coalition.	*National organisation representing rural women*	429
SA Birth Matters.	*Consumer group (natural birth)*	187
Hunter Home and Natural Birth Support	*Consumer group representing home birth*	71
Future Families	*New mother support group*	74
Laynhapuy Homelands Association INC	*Indigenous elders*	211
Consumer Advisory Council (CAC) of King Edward Memorial Hospital, Perth	*Consumer representatives of tertiary maternity hospital*	223
Northern Territory Health and Community Services Complaints Commission	*National organisation representing women*	240
Birthrites: Healing after caesarean section Inc	*Community support organisation*	572
Health Care Consumers Association	*National organisation of health care consumers*	324
Birth Healing	*National online consumer support forum*	703
Key Centre for Women's Health in Society	*Women's health research centre*	369
Birth and Beyond	*Consumer group*	456
Mother & Child Health Research	*Interdisciplinary research centre*	468
Mums@ Ryde	*Consumer group (natural birth)*	662
Country women's Association NSW	*Rural organisation representing women*	683
Women's Health West	*Organisation representing migrant women*	410
Bush Babies	*Parent group*	293
Friends of Mackay birth centre	*Birth centre consumer group*	297
N	*Mother of 1. Private hospital*	16
P	*Mother of 2. Public hospital*.	534
R	*Mother of 5. Public then home birth × 1*	783
S	*Mother of 2 (twins). Public hospital*	693
M	*Mother of 1. Public hospital*	673
C	*Mother of 2. Private hospital then birth centre*	653
Unidentified	*Mother of 3.Private hospital × 2 then home birth*	523
Unidentified	*Mother of 5. Public hospital × 3 Home birth × 2*	483
J	*Mother of 3.Birth centre*	300
N2	*Mother of 1. Birth centre/home*	093
H	*Mother of 2. Birth centre then home*	730
G	*Mother of 5. Private, Public hospital then *	603
V	*Mother of 3. Public hospital then home × 2*	513
J	*Mother of 2. Public hospital then home birth*	413
A	*Mother of 2. Public hospital then home birth*	203
S2	*Mother of 4. Rural*	30

http://www.health.gov.au/internet/main/publishing.nsf/Content/maternityservicesreview-submissions

Methodological rigour in this study was achieved in the provision of a full explanation of the relationship between the discourse and results in addition to providing textual evidence to support these results. A full description of the rationale for inclusion of each submission is provided in Table 1.

### Data Analysis

The particular methodology utilised when analysing discourse varies according to the nature of the research question and the particular discursive practice under examination [24]. Fairclough (2003) described a three step process when undertaking discourse analysis. The analytic framework informing the critical analysis of text in this study included the following step by step processes; becoming familiar with the text, identifying themes and examining relationships between discourses, identifying discursive strategies used to sustain the discourse and examining the effects of the discourse. The first step included the following questions: who is speaking and on behalf of who; who are the key stakeholders and who is silent. Analysis of the text in step 2 comprised the following questions: who is the subject of the particular discourse; what position is promoted by the discourse and in whose interest is this position; what are the hidden agenda's/biases; what sorts of decisions have been made and with what level of authority and influence. The third step of data coding used the codes identified in steps 1 and 2 to search for instances where power and knowledge were present in the discourse and employed a deductive approach. Analysis of the text in step 3 was based on the following questions: what power-knowledge relationships exist; who are the people with the power to make decisions and how are they using their power [24].

## Results

Critical analysis of selected submissions (N = 36) to the national review of maternity services in 2008 by mothers, consumer support groups and groups representing women on this issue identified a number of discourses exerting influence on the primary maternity care reform process. These discourses will be discussed under the headings derived from a content analysis of the submissions included in this study. The headings include: hospital birth; rural setting; birth centre; homebirth and the reform.

Submissions from consumer groups presented a unified position. The current model of maternity care is deemed to be unnecessarily costly and associated with a negative impact on the health of women [35-37]. Discourses generated in this study from analysis of the submissions from the maternity coalition (MC), SA birth matters and the multicultural centre for women's health [35-37] included the following: 'The interests of women and their babies must be the focus of maternity care, not the system and those who work in it'. This discourse implied that current maternity services operate in the interests of the institution and maternity care professions, not in the interests of the consumers receiving care. 'Living in rural communities should not deny women the right to the same quality of maternity care and access as their city sisters'. This discourse highlights inequity embedded in a system that privileges one group over another, usually more vulnerable group. 'Maternity care options need to be expanded to allow women choice in the care they receive'. This discourse infers that women are not being offered choice in the maternity care they elect to receive. 'Rates of medical intervention in Australia are too high', a discourse that challenges current obstetric practices employed or healthy pregnancies.

### Hospital birth

The majority of Australian women in 2008 (96.9%) are reported as giving birth in either obstetric -led public or private hospitals [23]. This trend reflects widespread acceptance across the country that birth needs to be medically managed. Specialised medical advancement has changed societies' expectations regarding childbirth. There is no longer need to give birth to a child with abnormalities as we have diagnostic tests to protect us. Reproductive technologies have rescued women from the consequences of infertility. Women no longer need to suffer the pain of labour as medical intervention makes this unnecessary, in the same way we no longer expect to experience pain in other areas of our lives [17]. Women want to avoid regret and in doing so place their own wellbeing secondary to that of the baby [17]. Authoritative knowledge is based on scientific evidence that is communicated by care providers. Non medical knowledge is devalued by all participants, usually including the woman herself who comes to believe that the course charted on the basis of professional medical knowledge is best for her [38]. Women and their partners accept medical intervention because they are afraid that something will go wrong and the perfect child is more important than the perfect birth [18].

Discourses identified include; 'we trusted the doctors to tell us everything we needed to know', 'we never questioned the doctor's advice on which maternity service was right for us', 'we did not know what we did not know'. Selected statements illustrating the meanings of these discourses included:

*'The first question the GP asks when pregnancy is confirmed is "do you have private health insurance?" If the answer is yes the GP will automatically refer to an obstetrician in a private hospital. It does not make sense that having private health insurance is the determinant in referring low risk healthy women to a highly trained specialist in complications' (submission 300)*.

*'The majority of women are not being given sufficient information to make an informed choice' (submission 662)*.

'Women lack knowledge of options in maternity care and are unable to offer their opinions or to exercise options' (submission 410)

*'I had no idea that going to a private hospital put me at higher risk of unnecessary intervention and caesarean section' (submission 653)*.

*'Replace the role of GP's (and obstetricians) where women discuss their pregnancy, receive information and make plans for pregnancy and birthing in a non medical way' (submission 74)*.

The rhetoric advocating the empowerment of women during childbirth through exercising their right to informed choice has backfired for many women who have found themselves labelled by the health professionals, on whom they rely, as being 'difficult' or 'untrusting' [18]. Discourses identified include; 'lost in a parade of strangers when all that is needed is one person to trust', 'modern medicine knows best and women are best served to listen to the experts'. 'Selected statements illustrating the meanings of these discourses included.

*'Women experience dismissive attitudes from health professionals towards their preferences and the use of procedures they would prefer not have to experience' (submission 369)*.

*'Medical staff use fear tactics to manipulate women into choosing the way they want them to ie your baby will die' (submission 483)*.

*'I was considered an imbecile despite all the knowledge I have accumulated' (submission 783)*.

*'I admit that labour is painful but it isn't stressful in itself. It is stressful being in the care of strangers' (submission 203)*.

*'Antenatal service at the public hospital was simply a testing service. It did not build a relationship with a midwife and all the testing kept me removed from my baby emotionally. I was too scared to begin a relationship with my baby in case something went wrong' (submission 413)*.

*'Women are often not included in decisions being made about their bodies and their babies. This leads to disempowerment, fear and distress' (submission 572)*.

Women expect childbirth to be a potentially non-affirming event steeped in pain and fear [39]. A small study of consumers undertaken in South Australia reported that birth experiences were at odds with the expectations of a significant number of women [22]. This finding was confirmed in a recent study of 141 Western Australian women's accounts of their birth experience with more descriptions of negative birth experiences than positive ones [25]. The act of giving birth to a child as considered a seminal life event for women [25]. Birthing safely is thought to be the most important thing a mother does for her child and safeguarding that child is implicit in this responsibility. Studies revealed that in the process of giving birth many women found themselves vulnerable and dependant; they put their trust in their care providers and willingly did whatever they were led to believe might help secure a healthy baby and control of birthing processes [18]. Fear of the unknown is an anticipated reaction for first mothers, a normal human reaction that fosters protection and safety in the species [40-43]. Of concern is when high levels of fear are sustained due to women's perception of their own birth risk being out of proportion to actual medical risk [40,44]. Communication of actual risk between women and health professionals is at fault. For example a woman may be told she has a 25% risk of developing a complication when it would be equally correct and more positive to state that she has a 75% chance of not developing the complication [41]. The ability to build a trusting relationship with the person caring for you has been a consistent theme throughout all the submissions to the review. Discourse generated include; 'the ability to form trusting relationships with a known carer reduces fear'. Selected statements illustrating the meanings of these discourses included:

*'It was devastating to be assigned in labour to a midwife who had actually left me in tears after an antenatal appointment. She had doubted anything in my carefully researched birth plan was allowed' (submission 783)*.

*'Continuity of care enables a pregnant women to develop a relationship with one person, ... and to build trust with that person. She knows that when it is time to labour, she will be professionally supported by someone who is aware of her history, understands her and cares for her and her family' (submission 662)*.

*'We would welcome innovative approaches to maternity service delivery that reduces episodes of fragmented care and enable women to have continuity of care with a known health professional' (submission 223)*.

*'Failure to focus care on women as individuals resulting in poor relationships with service providers' (submission 410)*.

Many women attempt to seek answers from caregivers when their expectations of their birth experience are not met. Fragmentation of service delivery and lack of a known carer impede women's ability to obtain the answers sought. In some cases the woman's aspirations regarding their desired birth outcomes are dismissed by health professionals as being unimportant. Some women in this situation find themselves treated as a complaining nuisance or a trouble maker, someone difficult to please. Discourse generated includes; 'at least you had a healthy baby'. Selected statements illustrating the meanings of this discourse include:

*'Many women, when telling others about their traumatic birthing experience, will be told: "At least you had a healthy baby". Whilst this statement is well intentioned, it is insensitive to the needs and emotions that birth trauma can entail' (submission 703)*.

*'I now know that the cone of silence that surrounds childbirth effectively silenced me from expressing my doubts, regrets and fears. I had a healthy baby and so, that was all that mattered' (submission 653)*.

*'I know it would have helped me greatly to be able to talk to someone before and after my caesareans to help me process my feelings of disappointment, instead of going on to suffer two and a half years of emotional upset' (submission 534)*.

### Rural Setting

Equity of access to maternity care in rural and remote communities was raised as a serious deficiency in current maternity service delivery [36], a situation exacerbated by continuing service closures [45]. Limited availability of services requires pregnant women to travel long distances for care due to closure of local services with many women needing to leave their community to give birth placing a substantial burden on families affected [45-47]. Discourse identified; 'women living in rural or remote communities have the same right to access safe maternity care close to home as their city contemporaries'. Selected statements illustrating the meanings of this discourse included:

*'I had to travel 160 kilometre round trip to the next town to see a Doctor I have never met before, in a town I don't frequent and give birth in a hospital far away from my family' (submission 30)*.

*'Once again, mothers/families are forced to accept service standard by postcode. Although they choose to live in remote or rural area's, they do not choose, nor should they be forced to accept second class citizenship status as is the case in maternity care' (submission 683)*.

*'There is a significant degree of concern, anger, frustration in the area of maternity and midwifery services which are seen to be inadequate and insufficient' (submission 429)*.

Of particular concern in remote communities was the situation indigenous women describe in their inability to access maternity care [48,49]. Statements describing the indigenous mothers experience in maternity care included:

*Indigenous women for whom English is a second language are transported alone from their community 2-4 weeks prior to the expected date of birth. This has been the practice for over 30 years. Why is it acceptable that an Aboriginal women make a journey away from her family without a chosen family member to support her? (Submission 211)*.

*'At present pregnant women living in remote areas are required to birth their babies in hospital. These women are often very young, alone and often birth alone' (submission 240)*.

*'Women are forced to leave their communities and travel to Perth-often unaccompanied-some weeks before their baby is due. This experience is extremely stressful for these women' (submission 223)*.

### Birth centre and home birth

The loudest voice in the consultation process came from supporters of natural birth representing birth centre and home birth options. Women unanimously reported being extremely satisfied with the care they received in all aspects of the birth experience. Independent practising midwives were revered for their ability to be trusted with the sacred privilege associated with being a participant in the birth of their child. Discourses included; 'safe and secure in a relationship built on mutual trust and respect'. Selected statements illustrating the meanings of this discourse were:

*'After my wonderful experience of giving birth to my second son which took place in my own bath tub, I believe strongly that being uninhibited and relaxed is of great value in labour. I find being watched in labour and birth very inhibiting. When women are able to form trusting relationships with a hands off' carer then the problems associated with inhibition are minimised and labour can proceed smoothly' (submission 730)*.

*'The support that our midwives have given us antenatally and while birthing has increased our positive feelings about motherhood, while their postnatal care has enabled us to feel bonded with and breastfeed our babies successfully' (submission 71)*.

*'Only women choosing home birth have true continuity of care' (submission 456)*.

Of interest a substantial number of women advocating for homebirth were casualties from a first birth experience in a public or private hospital. These women sought a different experience and provide comparisons of their distressing experience of hospital care with their rewarding experience of giving birth naturally at home with their subsequent baby.

*'The central reason for choosing to have a homebirth with my 2^nd ^baby was how appalling our experience was in the public hospital' (submission 513)*.

*'My home birth served to re-empower me as a woman. I felt cared for, supported, nurtured. I was not a failure!! My son and I were treated as a holistic unit, mother and child. It was so beautiful!!!' (submission 783)*.

In these cases loss of trust in the maternity care professionals who failed to rescue them at their most vulnerable time, feature strongly in their accounts [50,51]. Discourse included; 'women need to be able to trust their care givers to act in their best interests, not in the interests of the hospital, doctors and midwives'.

*'Firstly because I wanted midwife-led care and I could not get that at my local hospital and second because I had already experienced unnecessary birth interventions previously and wanted to avoid that again' (submission 523)*.

' When I discovered my name did not make the ballot for the birth centre I felt distressed and grieved that I would by default now have to give birth in a hospital. Home birth was the only alternative available to me' (submission 93)

### The reform

A range of recommendations for maternity care reform was provided in the submissions to the review from organisations representing the position of women. The discourse identified; 'one size will not fit all' is a reminder that women are not a homogenous group when it comes to the type of maternity care that will best suit their needs. Selected statements illustrating the meanings of this discourse included:

*'Different women have different needs in relation to pregnancy, childbirth and motherhood and therefore considers that no one model of service delivery is suitable across Australia and that flexibility is essential' (submission 220)*.

*'Women need to have access to maternity services that are appropriate to their clinical, cultural and social needs'. Giving birth is a life event rather than a medical event and the health system needs to take a broader view when planning service to meet our needs' (submission 324)*.

*'A known care provider to be chosen by the women may be a GP, obstetrician, publicly funded midwifery led service or independent midwife in private practice' (submission 293)*.

*'Midwifery led pregnancy and birth care has been proven to lead to safe, positive outcomes in well, healthy women. And we believe that this model of care should be available as an option for all pregnant women' (submission 297)*.

*'There is evidence to support General Practitioner (GP) models of maternity care as satisfactory for women and just as safe when compared to obstetric-led care' (submission 468)*.

Recommendations for reform throughout the submissions selected in the study also highlighted the need to resolve the contentious issue of vaginal birth after caesarean section (VBAC). Rising rates of emergency caesarean sections are typically associated with a prolonged first labour combined with inadequate analgesia resulting in an exhausted mother [52]. Women often feel betrayed by maternity care professionals when they find that because they have succumbed to caesarean section for their first baby, they no longer have the option to birth vaginally with future babies. Giving birth is seen as a life affirming event where women unable to live up to their own expectations of the first birth are driven to do better next time [25]. This opportunity denied the increasing numbers of women who unknowingly found themselves caught up in the 'one caesarean always caesarean' trap.

'I felt I was being bullied into having a repeat caesarean-The reasons the doctor was giving were more to do with convenience for him and the hospital. I was distraught at this but got no compassion from my obstetrician who by this stage was treating me as if I was being difficult' (submission 534)

*'My pregnancy was trouble free so I was shocked and very upset when 5 days before my due date my obstetrician told me I would have to be booked in to have a caesarean. My daughter was born the next day by "elective caesarean" (I use the term elective unwillingly-I did not elect it, my doctor did)' (submission 534)*.

'Consideration for a "Next birth after caesarean clinic" with a view to supporting women who wish to choose a vaginal birth after a previous caesarean birth' (submission 223)

## Discussion

The discourses identified and described in this critical analysis of selected submissions to the national review of maternity services are clearly reflected in the proposed primary maternity care reforms. The notable omission in the data was the absence of submissions from women satisfied with their obstetric -led maternity care. The silence from this group throughout the consultation process has resulted in a concentration of consumers advocating for a move away from obstetric -led care in healthy pregnancy. The silent consumer voice has diminished the position of advocates for obstetric -led maternity care in a climate of rising consumer participation. The rise of consumer influence reflects a changing of the guard as doctors' traditional authority is questioned by strong consumer organisations and informed consumers [53]. The powerful consumer influence can be seen as a feature of a neoliberal approach to health management encapsulated by a corporate governance ethos [54]. Consumerism is a central discourse within corporate governance [54] and when applied to maternity services promotes consumer choice and individual autonomy [22]. Consistent with these values women are encouraged in numerous websites hosted by the Australian government to empower themselves through informed decision making regarding maternity care [55]. The consumer discourses identified in this study; 'the interests of women and their babies must be the focus of maternity care, not the system and those who work in it', and 'maternity care options need to be expanded to allow women choice in the care they receive' are reflected in the primary maternity care agenda for change. However the opposing discourses 'modern medicine knows best and women would be best served to listen to the experts' and 'at least you have a healthy baby' convey a very different message positioning women seeking non obstetric -led care options at odds with maternity care providers.

The discourses of women living in rural and remote communities and strong consumer organisations representing the interests of these women have featured strongly in the proposed reform agenda [45,48,49,56]. The spotlight on the shameful plight of indigenous women living in remote communities being expected to leave home unescorted by a loved one to give birth in a foreign environment has been catalytic in support for primary maternity care reform [48,49]. Returning low risk maternity services to local communities is a feature of the change agenda [5]. The discourse 'living in rural communities should not deny women the right to the same quality of maternity care close to home as their city contemporaries' is synergistic with good corporate governance committed to equity of access. The discourse highlights the ongoing inequity in the current maternity care system that must be addressed.

Women from all geographic areas reported a lack of availability of non obstetric- led services where demand for existing services is greater than service capacity [36]. A number of women chose home birth when faced with the prospect of a subsequent hospital birth. A traumatic birth experience was a common theme amongst a sizeable proportion of women choosing home birth for their second or subsequent birth. The home birth experiences were universally positive in contrast to the previous birth in a high tech obstetric service. The discourses 'women need to be able to trust their caregivers to act in their best interests, not in the interests of the hospital, doctors or midwives' and 'safe and secure in a relationship built on mutual trust and respect with a known carer', represent the differences in experience between a fragmented maternity service and what can be achieved in a caseload continuity of care model.

It appears that women are becoming more fearful of the birthing process than ever before, a situation compounded by feelings of vulnerability in relationships when confronted with a constant stream of unknown maternity care professionals [42]. The benefits afforded women in labour associated with a relationship based on trust with a known caregiver feature strongly throughout the submissions [48,57]. The ability to build a relationship with a known carer based on mutual respect and trust is promoted by advocates of natural birth as the panacea for being able to control levels of fear, maintain feelings of being in control throughout the birthing process and being satisfied with the outcome of the experience [42]. Formation of trusting relationships requires regular interactions between the pregnant woman and the known carer, only achievable in continuity of care models a key element in the proposed primary maternity care reforms. The discourses 'not knowing what we don't know' and we trusted the doctors to tell us everything we needed to know' refers to the total trust that women pregnant for the first time and their partners place on obstetric-led maternity care. The discourse 'lost in a parade of strangers when all we needed was one person to trust' captures the consumer experience of a fragmented maternity service care and subsequent distress associated with finding themselves in territory they never dreamed possible [22].

A strong theme throughout the submissions related to the controversy of vaginal birth after caesarean section (VBAC). Consumer groups representing the interests of women across a range of areas are united in the call for reconsideration of obstetric practices that deny women the opportunity to experience a trial of labour in the subsequent birth, rather than automatically opting for an elective caesarean section [37,56,58-60]. It is argued that the majority of medical indications for emergency caesarean section in the first birth do not extend to subsequent births [58] and that obstetric practices denying women the option of a trial of labour are based on the interests of health professionals and the organisation rather than those of the woman caught in this situation [49]. It is a concern to find that women whose interests would be best serviced by specialist support during attempted VBAC are being turned away from these services to home birth. Advocates of home birth report a growing number of resourceful women birthing at home under the care of independent midwives achieving a successful VBAC [61].

There is strong support for the proposed reforms from women who participated in the consultation process. However the AIHW Australia's mothers and babies series for the year 2008 provides evidence in support of the presence of another view that is not represented in this study [23]. The silent view pertains to the notion that many women are getting what they want from their obstetric -led maternity care as illustrated by the speed in which Australian society has accepted the ability to take a 'taxi to the finish line' by way of elective caesarean section birth [18] and illustrated by the following statement.

*'It is our experience that women who choose to birth by caesarean section can have more empowered and positive experiences when that decision is well informed and not based on fear' (submission 572)*.

Consumer organisations representing the interests of all women accessing maternity care advocate strongly for an increased range of maternity care options [37,59]. In doing so these organisations are representing the silent voice of women who were satisfied with their obstetric-led care and those who may wish to choose this option in the future. The discourse 'one size will not fit all' reminds us that women, reflecting the communities in which they live, have different belief systems when it comes to their choice of maternity care and the right to informed choice must be supported [59,60]. The primary maternity care reform agenda must retain enough flexibility to meet the maternity care needs of all Australian women.

## Conclusion

Findings from this critical discourse analysis revealed extensive similarities between the discourses identified in the submissions by individual women, consumer groups and other organisations representing women's interests with the direction of the proposed primary maternity care reform agenda. Unified consumer influence advocating a move away from obstetric -led maternity services for all, toward primary maternity care, appear to be synergistic with the ethos of corporate governance and a neoliberal approach to maternity service policy. The silent voice of one consumer group in the consultation process has inadvertently contributed to a consensus of opinion in support of the reforms in the absence of the counter viewpoint.

## Competing interests

The authors declare that they have no competing interests.

## Authors' contributions

MM was responsible for the study conception, design, review and analysis of the literature and drafting of the manuscript. KF and YC made critical revisions to the paper for important intellectual content. All authors have approved the final manuscript.

## Pre-publication history

The pre-publication history for this paper can be accessed here:

http://www.biomedcentral.com/1471-2393/11/53/prepub
